# TRAPP Complexes in Secretion and Autophagy

**DOI:** 10.3389/fcell.2016.00020

**Published:** 2016-03-30

**Authors:** Jane J. Kim, Zhanna Lipatova, Nava Segev

**Affiliations:** ^1^Department of Biological Sciences, University of Illinois at ChicagoChicago, IL, USA; ^2^Department of Biochemistry and Molecular Genetics, University of Illinois at ChicagoChicago, IL, USA

**Keywords:** Ypt, Rab, GTPase, GEF, secretion, autophagy, tether

## Abstract

TRAPP is a highly conserved modular multi-subunit protein complex. Originally identified as a “transport protein particle” with a role in endoplasmic reticulum-to-Golgi transport, its multiple subunits and their conservation from yeast to humans were characterized in the late 1990s. TRAPP attracted attention when it was shown to act as a Ypt/Rab GTPase nucleotide exchanger, GEF, in the 2000s. Currently, three TRAPP complexes are known in yeast, I, II, and III, and they regulate two different intracellular trafficking pathways: secretion and autophagy. Core TRAPP contains four small subunits that self assemble to a stable complex, which has a GEF activity on Ypt1. Another small subunit, Trs20/Sedlin, is an adaptor required for the association of core TRAPP with larger subunits to form TRAPP II and TRAPP III. Whereas the molecular structure of the core TRAPP complex is resolved, the architecture of the larger TRAPP complexes, including their existence as dimers and multimers, is less clear. In addition to its Ypt/Rab GEF activity, and thereby an indirect role in vesicle tethering through Ypt/Rabs, a direct role for TRAPP as a vesicle tether has been suggested. This idea is based on TRAPP interactions with vesicle coat components. While much of the basic information about TRAPP complexes comes from yeast, mutations in TRAPP subunits were connected to human disease. In this review we will summarize new information about TRAPP complexes, highlight new insights about their function and discuss current controversies and future perspectives.

## Introduction

Trafficking between intracellular compartments is mediated by vesicles and regulated by the highly conserved Ypt/Rab GTPases, their nucleotide exchangers, GEFs, and their downstream effectors (Lipatova et al., 2015a). Multi-protein complexes facilitate vesicle formation at donor compartments, subsequent targeting, tethering and fusion with acceptor compartments. One such complex, the transport protein particle TRAPP, acts as a Ypt/Rab GEF and possibly as a tether.

Yeast TRAPP was identified as a ten-subunit protein complex (~800 kDa) that co-precipitated with Bet3, which mediates endoplasmic reticulum (ER)-to-Golgi transport (Sacher et al., 1998, 2000). To date, eleven TRAPP subunits conserved between yeast and humans have been identified. Seven subunits are small, 140–283 amino acids, and the rest are larger, 417–1435 residues. These subunits are termed TrsN (*N* = size in kDa) or Bet3/5 in yeast, and TrappC (or TCP) 1–10 and 13, in mammalian cells. In yeast, these subunits assemble into three known complexes, TRAPP I, II, and III. Six of the small subunits form TRAPP I or attach to it for assembly of the two other complexes, while not much is known about the seventh small subunit Tca17. The large subunits are exclusive to either TRAPP II or TRAPP III complexes. Two additional subunits, TrappC/TCP 11-12, are metazoan specific and their TRAPP-related function will be discussed (Table 1).

**Table 1:** **TRAPP complexes Subunits**.

**Subunit Group**	**Yeast Trs/Bet (aa)^a^**	**Human TrappC/TPC (*alias*) (aa)^a^**	**Comments**	**Disease Connections (References for recent additions)^b^**
Core TRAPP	Bet5 (159)	TrappC1 (*Mum2*) (145)	Ypt1 binding	Cancer
	Bet3 (193)	TrappC3 (180)		Not found^c^
	Trs23 (219)	TrappC4 (*Synbidin*) (219)	Ypt1 binding	Carcinogenesis (Weng et al., 2014)
	Trs31 (283)	TrappC5 (188)		Not found^c^
TRAPP I-associated	Trs33 (268)	TrappC6 a,b (159, 158)		Alzheimer's Disease (Hamilton et al., 2011; Chang et al., 2015)
Adaptor	Trs20 (175)	TrappC2 (*Sedlin*) (140)	Interacts with Trs85 & Trs120	SEDT, Miscarriage (Wen et al., 2015)
	Tca17^d^(152)	TrappC2L (140)		Not found^c^
TRAPP III-specific	Trs85 (698)	TrappC8 (1435)		Required for HPV entry (Ishii et al., 2013)
TRAPP II-specific	Trs120 (1289)	TrappC9 (*NIBP*) (1148)		Cancer (Zhang et al., 2015); Intellectual disability (Khattak and Mir, 2014); schizophrenia (McCarthy et al., 2014)
	Trs130 (1102)	TrappC10 (*TMEM1*) (1259)		Cancer (Pongor et al., 2015)
	Trs65 (560)	TrappC13 (*C5orf44*) (417)	Yeast TRAPP II assembly, dimer formation	Not found^c^
Metazoan	NA	TrappC11 (*C4orf41*) (1133)	mTRAPP III^e^	Muscular dystrophy
	NA	TrappC12 (*TCC-15*) (735)	mTRAPP III^e^/Moonlighting in mitosis	Not found^c^

a *(aa): number of amino acids*.

b *Previous disease connections are summarized in Brunet and Sacher (2014)*.

c Disorders Not found: as of November 2015 in “Gene Cards database.”

d *Tca17 is grouped with its closest paralog Trs20 even though its role as an adaptor has not been shown*.

e *Mammalian TRAPP III*.

Based on similarities between the purified Ypt1 GEF (Jones et al., 1998) and TRAPP, the activity of TRAPP as a Ypt GEF was determined (Jones et al., 2000; Wang et al., 2000). The idea that TRAPP acts as a tether to mediate vesicle docking was proposed at the same time based on the requirement of TRAPP for association of ER vesicles with the Golgi in a cell-free system (Barrowman et al., 2000). Since 2000, while evidence has accumulated regarding the importance of TRAPP subunits in human health (Brunet and Sacher, 2014), many uncertainties and controversies about the mechanisms of action of the TRAPP complexes have piled up.

Here, we summarize current views on TRAPP complexes structure, their function as Ypt/Rab GEFs and tethers, and their connection to human disease. Most of the evidence on the structure and function of TRAPP complexes comes from yeast and pertains to mammalian cells, while studies of human TRAPPs highlight their importance to human health.

## TRAPP complexes: Composition, structure, and architecture

### TRAPP complex composition

In early reports about TRAPP, ten of the eleven yeast TRAPP subunits, except for Tca17, were found in a single high-molecular weight complex (~800 kDa) that co-precipitated with tagged Bet3 (Sacher et al., 2000). Later, two complexes I and II, were separated by gel filtration chromatography, where the larger complex (~1000 kDa) contains all the subunits of the smaller complex (~300 kDa), plus three large subunits (Sacher et al., 2001). Similarly, all the mammalian TRAPP subunits can be purified as a large complex (~670 kDa) by co-precipitation with a single core TRAPP subunit (Yamasaki et al., 2009).

The problem with assigning individual subunits to a specific TRAPP complex based solely on their purification from cell lysates is that TRAPP complexes oligomerize in cell lysates (Choi et al., 2011). The fact that this oligomerization depends on salt concentration or detergents suggests that it happens during cell lysis or purification (Brunet et al., 2012, 2013). Homotypic or heterotypic oligomerization can explain why the apparent molecular weights of TRAPP I and TRAPP II are higher than their expected sizes based on their composition; e.g., the expected size of TRAPP I is ~170 kDa, the size of recombinant TRAPP I purified from bacteria is ~170 kDa (Kim et al., 2006) as compared to ~300 kDa for the complex purified from yeast (Sacher et al., 2001). Oligomerization can also explain the observation that Trs85 fractionates with all the other TRAPP subunits (Sacher et al., 2000) even though it does not seem to function with TRAPP II. Therefore, assignment of individual subunits to specific TRAPP complexes should be based not only on their presence in purified complexes, but also on *in vivo* assays such as phenotypic and co-localization analyses.

Currently, in yeast all the small subunits are assigned to TRAPP I (Sacher et al., 2008). However, even though a recombinant yeast TRAPP I complex can be purified with six small subunits in stoichiometry of 2:1 Bet3 to each of the other five subunits (Kim et al., 2006), it is not clear whether two of these subunits, Trs20 and Trs33, function as part of this complex. Trs20 plays a role as an adaptor and is required for the assembly of the large TRAPP complexes: the association of TRAPP I with Trs120 for TRAPP II assembly (Taussig et al., 2013), and with Trs85 for TRAPP III assembly (Brunet et al., 2013; Taussig et al., 2014). However, it is currently unknown whether Trs20 is required for the function of TRAPP I. Tca17, a paralog of Trs20, also does not have a known function with TRAPP I (see below). Unlike the other small TRAPP subunits, Trs33 is not required for yeast cell viability or secretion. A role for Trs33 in the context of TRAPP I is currently unknown, and it is required for TRAPP II assembly only when Trs65 is absent (Tokarev et al., 2009). Therefore, in Table 1, Trs33 is listed as a TRAPP I-associated subunit. TRAPP I cannot be separated from the mammalian TRAPP II and its existence *in vivo* in mammalian cells is currently in question (Barrowman et al., 2010).

The assignment of Trs120, Trs130, and Trs65 as TRAPP II-specific subunits in yeast is based on their purification with this complex and on mutational analysis (Sacher et al., 2001; Liang et al., 2007). Trs85, in spite of its co-purification with TRAPP I, is considered as a TRAPP III-specific subunit in yeast (Sacher et al., 2008).

A single TRAPP complex purified from mammalian cell lysate contains all the known subunits, including the Trs85 ortholog, TrappC8 (Scrivens et al., 2011). However, based on genetic interaction maps, two TRAPP complexes were suggested to exist in mammalian cell: TRAPP II, which contains core TRAPP plus TrappC9-10, and TRAPP III, which contains core TRAPP plus TrappC8, 11-13 (Bassik et al., 2013). The inclusion of TrappC13, the homolog of Trs65, in the mammalian TRAPP III differs from its inclusion in the yeast TRAPP II complex. Importantly, there is currently no evidence for the existence of TRAPP I in mammalian cells.

#### Identifying new TRAPP subunits

A number of proteins were suggested to be TRAPP subunits. Tca17 (TRAPP complex associated) and its mammalian ortholog TrappC2L (TrappC2-like) were identified as sub-stoichiometric components of purified TRAPP that have sequence similarity to Trs20/Sedlin, and may play a role in in TRAPP assembly or stability (Montpetit and Conibear, 2009; Scrivens et al., 2009). Two metazoan-specific subunits, TrappC11, which is distantly related to Trs130/TrappC10, and TrappC12, were identified based on their co-fractionation with TRAPP and effect of their depletion on ER-to-Golgi transport (Wendler et al., 2010; Scrivens et al., 2011). A role for TrappC12, renamed TRAMM, without the rest of the TRAPP complex, was recently shown in mitosis (Milev et al., 2015). While it is possible that TrappC12 has a moonlighting function in mitosis, it suggests that proteins that associate with TRAPP are not necessarily TRAPP subunits. Therefore, additional criteria should be considered before assigning a Trs or TrappC number for new yeast and mammalian subunits, respectively.

### TRAPP subunit structure

The crystal structure of all the small yeast and mammalian TRAPP subunits has been resolved. Structurally, the subunits fall into two groups. Members of the Bet5 group, which also includes Trs20, Trs23, and Tca17, have a longin-domain (LD) fold (α–β–α sandwich), which is involved in protein-protein interactions and was suggested to form a LD-LD platform for GTPase binding (Levine et al., 2013). Specifically, in TRAPP I, Bet5 and Trs20 form a LD-LD platform for Ypt1 binding (Cai et al., 2008). Members of the Bet3 group, which also includes Trs31 and Trs33, have similar structures (α/β-plait topology) despite low sequence similarity (Kümmel et al., 2005), and hetero-dimerize with Bet3 in the context of TRAPP I (Kim et al., 2006). Currently, the structures of the large TRAPP subunits are not resolved.

### TRAPP complex architecture

**Figure 1:**
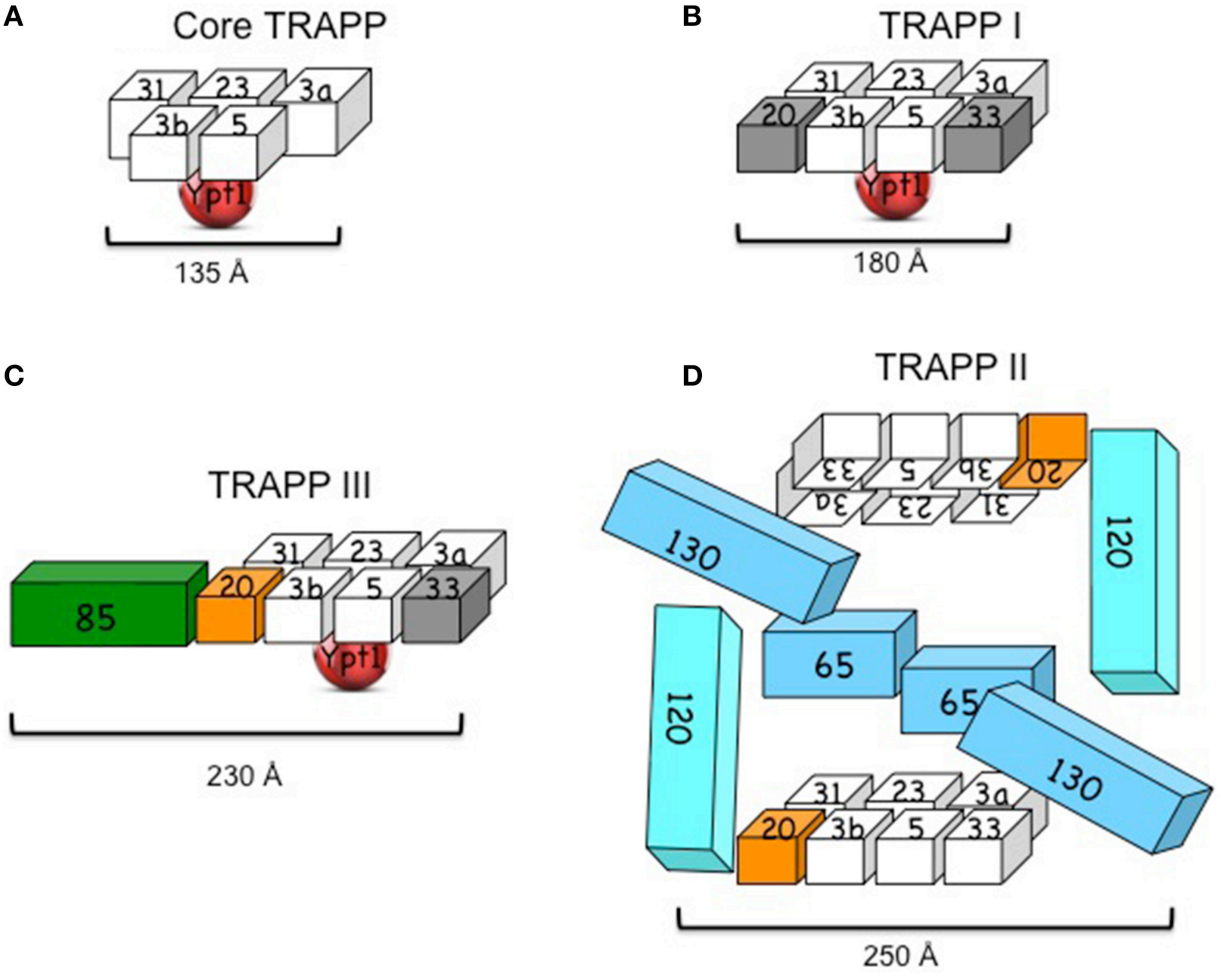
**Yeast TRAPP complexes. (A)** Core TRAPP, which contains four small subunits with two copies of Bet3 (3a and 3b), self assembles, binds Ypt1 through Bet5 and Trs23 and acts as a Ypt1-GEF. The diagram is based on the published structure of core TRAPP in a complex with Ypt1 (Cai et al., 2008). **(B)** TRAPP I contains core TRAPP, Trs20 and Trs33. However, it is not clear that the presence of the latter two subunits (in gray) is required for its function. The diagram is based on the published EM structure of core TRAPP subunits with Trs33 or Trs20 (Kim et al., 2006). **(C)** TRAPP III contains core TRAPP, Trs20 (orange), which is required for its assembly with Trs85 (green) and its function in autophagy. A role for Trs33 (in gray) in this complex has not been shown. The diagram is based on the published architecture and mutational analysis of TRAPP III (Tan et al., 2013; Taussig et al., 2014). **(D)** TRAPP II contains TRAPP I, including Trs20 (orange) and Trs33, and three large subunits: Trs120, Trs130, and the non-essential Trs65. Trs20 is required for the interaction of TRAPP I with Trs120, and either Trs65 or Trs33 are required for assembly of TRAPP II *in vivo*. TRAPP II is depicted here as a dimer (see discussion in the text). The cubes representing the subunits are roughly proportionate to their size; numbers stand for TrsN, except for Bet3 and Bet5. Approximate length (Å) is based on crystal structure for core TRAPP and negative staining EM for the other complexes (see text).

While the subunit organization of TRAPP I was solved by crystallography, the architecture of the larger complexes was suggested based on single-particle electron microscopy (EM) and mutational analyses (Figure 1).

#### TRAPP I architecture

Recombinant core yeast TRAPP complex can self assemble when at least four subunits are co-expressed in bacterial cells: Bet3, Bet5, Trs23, and Trs31 (Table 1). The three-dimensional structure of core TRAPP has been solved by crystallography. The ~135 Å-long and 35–70 Å-wide cylinder contains two copies of Bet3 and one copy of each of the other three subunits (Cai et al., 2008). Two additional small subunits, Trs33 and Trs20, can attach at the two ends of the yeast core TRAPP, if co-expressed with the core subunits, to form the six-subunit TRAPP I. This complex is ~180 Å long and 65 Å wide. An atomic model for subunit organization of TRAPP I was suggested by single particle EM analysis and docking of crystal structures of two sub-complexes of mammalian TRAPP I. The two Bet3 copies have similar orientations to the same flat side of the complex, which contains both acidic and basic patches and is thought to face the membrane (Kim et al., 2006). Ypt1 binds the opposite, mostly acidic, side of TRAPP I (Kim et al., 2006; Cai et al., 2008).

#### Yeast TRAPP III architecture

The architecture of the yeast TRAPP III was suggested based on negative EM staining and docking of the atomic model of TRAPP I (see above) in the EM density map of TRAPP III. According to this model, Trs85 attaches to TRAPP I through Trs20, to form an elongated crescent shaped complex with approximate dimensions of 230 × 68 × 46 Å. Ypt1 binds to the same position as in TRAPP I (Tan et al., 2013). The EM structure is supported by mutational analysis of Trs20, which identified the Trs20 interface where Trs85 attaches to TRAPP I (Taussig et al., 2014). However, the structure of Trs85 and its interface with TRAPP I are not known.

#### TRAPP II architecture

The architecture of TRAPP II purified from yeast cell lysates was proposed based on single-particle EM and docking the atomic model of TRAPP I (see above). The suggested ~225 × 250 Å diamond-like structure is of a dimer in which the TRAPP II-specific subunits are sandwiched between two anti-parallel TRAPP I complexes (Yip et al., 2010). Currently, there is no evidence that dimerization of TRAPP II is required for its role in secretion to maintain cell viability, since Trs65, which is required for this dimerization, is not essential for cell viability. Moreover, in this model, the assignments of Trs120 and Trs130 to the Trs33 and Trs20 sides of TRAPP I, respectively, do not agree with existing mutational analyses. We have found that whereas Trs20 is required for the attachment of Trs120 to TRAPP I, Trs33 plays a role in the interaction of Trs130 with TRAPP I (Tokarev et al., 2009; Taussig et al., 2013), thus placing Trs120 and Trs130 to the opposite sides of TRAPP I when compared to the EM-based structure (Figure 1D). Considering these disagreements, the uncertainty regarding the significance of the dimer, and the fact that structural information is currently not available for the large subunits of TRAPP II, we conclude that the architecture of TRAPP II is still unclear.

## TRAPP complexes cellular function

### Yeast TRAPP I-III

**Figure 2:**
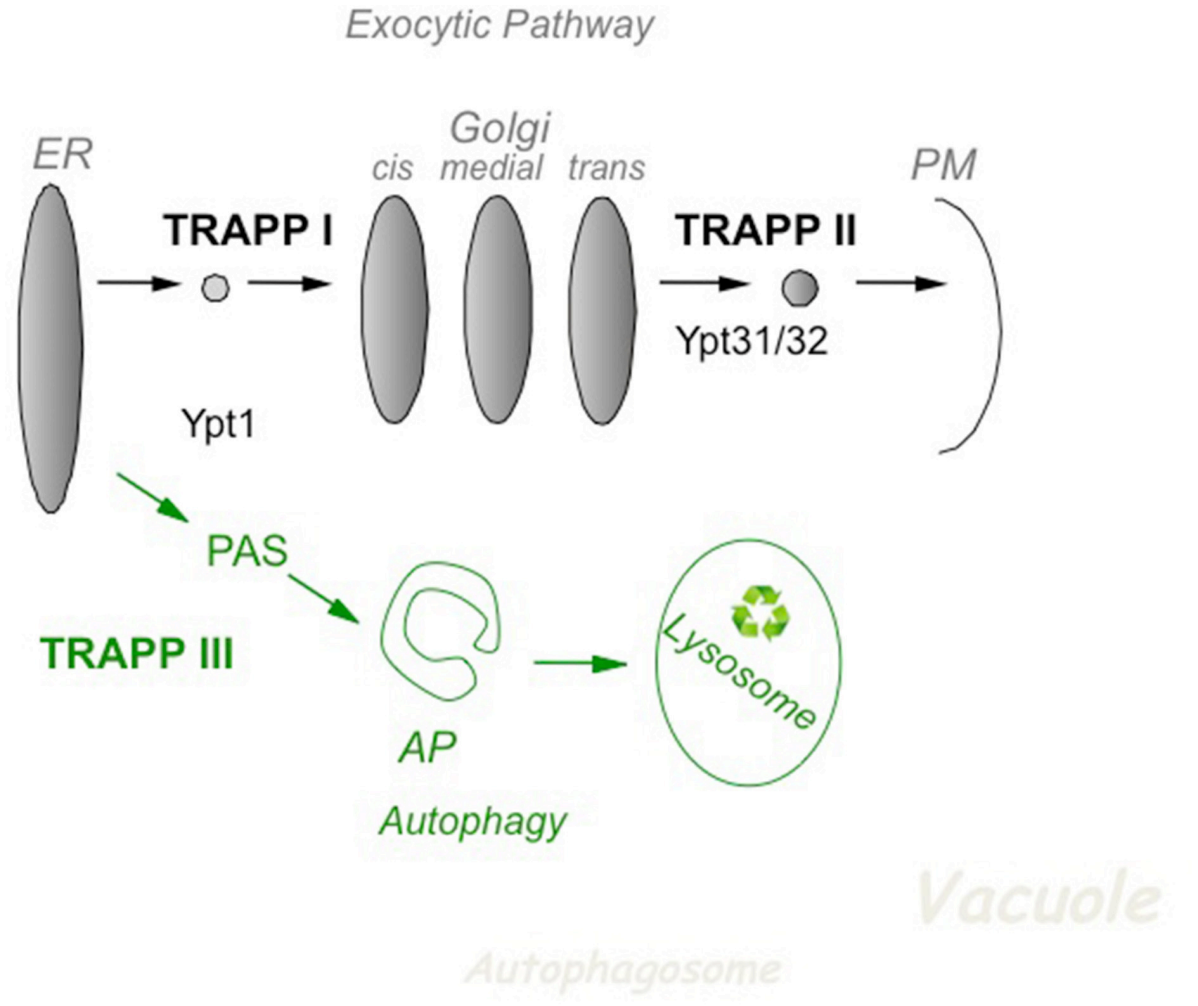
**The role of TRAPP complexes and their Ypt substrates in yeast intra-cellular trafficking**. In the exocytic pathway (top), TRAPP I activates Ypt1 to regulate ER-to-Golgi transport, whereas TRAPP II activates Ypt31/32 to regulate Golgi-to-PM transport. In autophagy (bottom), a cellular recycling pathway (in green), TRAPP III activates Ypt1 to regulate the assembly of PAS, the first step of autophagy. PAS is required for the formation of the double-membrane autophagosome (AP), which delivers cargo for degradation in the lysosome. See discussion in the text.

In yeast, based on effect of mutations in subunits of TRAPP I, II and III, it is now accepted that TRAPP I acts at early Golgi, TRAPP II in late Golgi, and TRAPP III in autophagy (Figure 2). The cellular function of the different TRAPP complexes can be inferred from whether they are essential for cell viability. In addition to the core TRAPP subunits, the Trs20 adaptor and the two TRAPP II-specific subunits are essential for cell viability, whereas the others, Trs33, Trs65, and Trs85, are not (Table 2).

**Table 2:** **TRAPP Subunit Essentially**.

**Subunit Group**	**Yeast Trs/Bet**	**Essential In Yeast**	**Human TrappC**	**Essential Hart et al^a^**	**Essential Blomen et al^b^**	**Essential Wang et al^c^**	**Essential in Human^d^**
Core TRAPP	Bet5	Yes	TrappC1	Yes (5/5)	Yes (2/2)	Yes (4/4)	Yes (11/11)
	Bet3	Yes	TrappC3	Yes (5/5)	Yes (2/2)	No (1/4)	Yes (8/11)
	Trs23	Yes	TrappC4	Yes (4/5)	Yes (2/2)	? (2/4)	Yes (8/11)
	Trs31	Yes	TrappC5	Yes (5/5)	? (1/2)	Yes (4/4)	Yes (10/11)
Others	Trs33	No	TrappC6A TrappC6B	No (0/5) No (0/5)	No (0/2) No (0/2)	No (0/4) No (0/4)	No (0/11) No (0/11)
	Trs20	Yes	TrappC2	(Not tested)	No (0/2)	No (0/4)	No (0/6)
	Tca17	No	TrappC2L	No (1/5)	No (0/2)	No (1/4)	No (2/11)
	Trs85	No	TrappC8	Yes (4/5)	Yes (2/2)	Yes (3/4)	Yes (9/11)
	Trs120	Yes	TrappC9	No (0/5)	No (0/2)	No (1/4)	No (0/11)
	Trs130	Yes	TrappC10	No (0/5)	No (0/2)	No (0/4)	No (0/11)
	Trs65	No	TrappC13	Yes (3/5)	No (0/2)	No (0/4)	No (3/11)
Metazoan	NA	NA	TrappC11	Yes (5/5)	Yes (2/2)	Yes (4/4)	Yes (11/11)
	NA	NA	TrappC12	No (0/5)	No (0/2)	No (0/4)	No (0/11)

a *Tested in 5 cell lines (number of cell lines in which the gene is essential/number of cell lines tested; Hart et al., 2015)*.

b *Tested in 2 cell lines (number of cell lines in which the gene is essential/number of cell lines tested; Blomen et al., 2015)*.

c *Tested in 4 cell lines (number of cell lines in which the gene is essential/number of cell lines tested; Wang T. et al., 2015)*.

d *Consensus: Yes, if gene is essential in at least 6/11 cell lines tested in the three studies*.

***Red:** Genes essential in yeast but not in human cells*.

***Green:** Gene essential in human cells, not in yeast*.

In the secretory pathway, cargo is delivered from the ER, through the Golgi apparatus, to the plasma membrane (PM). A set of Sec proteins that mediate the different steps of this pathway are essential for cell viability (Feyder et al., 2015). The essential TRAPP subunits make up the two TRAPP complexes, I and II, which function in the secretory pathway. The assignment of TRAPP I and TRAPP II to early and late Golgi, respectively, is based on mutation analysis. Specifically, whereas conditional mutations in Bet3 and Bet5, which are present in all TRAPP complexes, elicit an ER-to-Golgi as the first blocked transport step, mutations in the TRAPP II-specific subunits Trs130 and Trs120 result in a late Golgi transport block (Sacher et al., 2001).

Autophagy is a cellular recycling process in which excess or damaged cellular components are delivered for degradation in the lysosome via a double-membrane organelle termed autophagosome (AP). This process starts with the formation of the pre-autophagosomal structure, PAS, by a set of ~40 autophagy-specific proteins, Atgs. Usually there is one PAS per cell. Atgs are not required for cell viability during normal cell growth, but only during stress, e.g., nitrogen starvation (Weidberg et al., 2011). The TRAPP III-specific subunit, Trs85, which is not essential for cell viability, was instead shown to play a role in selective and non-selective autophagy (Meiling-Wesse et al., 2005; Nazarko et al., 2005).

While currently it is not clear how TRAPP complexes localize to the compartment on which they function, Golgi or AP, there are conflicting reports about the relationship between Trs85 and Atg9 and localization to PAS. Atg9 is the only integral membrane Atg and Atg9 vesicles are considered to bring the membrane component of the PAS (Weidberg et al., 2011). Trs85 and Atg9 co-localize on PAS, and also in multiple puncta in the cytosol (Lipatova et al., 2012). Based on co-immuno-precipitation and yeast-two hybrid interaction of Trs85 and Atg9 (neither shows a direct interaction), and the finding that Trs85 is not recruited to PAS in *atg9*Δ mutant cells, it was suggested that Atg9 brings TRAPP III to PAS (Kakuta et al., 2012). While this is a possibility, the problem is that in *atg9*Δ mutant cells there is no membrane in PAS and therefore no place for Trs85 or TRAPP to attach to. In contrast, it was proposed that TRAPP III plays a role in bringing Atg9 to PAS (Shirahama-Noda et al., 2013). Hopefully, future experiments will help resolve this issue.

### Mammalian Golgi TRAPP

In yeast, TRAPP subunits required for secretion are essential for cell viability. In three recent screens of ~20,000 human genes, about 2000 essential genes were identified (Blomen et al., 2015; Hart et al., 2015; Wang T. et al., 2015), with about a third having yeast essential orthologs (Blomen et al., 2015). We looked for the essentiality of TRAPP subunits in these three studies, which together analyzed 10 cell lines in 11 independent gene-trap or CRISPR/Cas9 screens (Table 2). The four core TRAPP complex subunits, TrappC1 (Bet5), TrappC3 (Bet3), TrappC4 (Trs23), and TrappC5 (Trs31), which are essential in yeast, are also essential in human cells. Two subunits not essential in yeast are also not essential in human cells: Trs33/TrappC6A/B (even though A and B could be supplementing for each other), and Trs65/TrappC13. The outliers are three genes essential in yeast but not in human cells: the adaptor Trs20/TrappC2, and the two TRAPP II-specific subunits, Trs120/TrappC9 and Trs130/TrappC10. In yeast, these three proteins assemble with core TRAPP to make TRAPP II, which is required for secretion. One subunit, Trs85, is not essential for yeast cell viability, whereas the human ortholog TrappC8 is; however, the similarity between these two subunits is very limited. Of the two metazoan-specific TRAPP subunits, one is essential, TrappC11, and one is not, TrappC12. Thus, more studies would be essential to interpret the reason for essentiality of the different TRAPP subunits in human cells.

Based on the migration of all the mammalian TRAPP subunits in a single high-molecular weight peak (~670 kDa) (Yamasaki et al., 2009), the currently dominating idea is that TRAPP II is the only mammalian Golgi TRAPP (Barrowman et al., 2010). However, because the expected size of individual TRAPP complexes is much smaller than the apparent ~670–800 kDa size of complexes purified from cell lysates, and because purified TRAPP complexes tend to oligomerize (Choi et al., 2011; Brunet et al., 2012), we suggest that the jury is still out regarding the existence of more than one mammalian Golgi TRAPP complex.

Mammalian TRAPP II was suggested to function in ER-to-Golgi transport based on the localization and knockdown effect of the TRAPP II-specific subunit mTrs130 (Yamasaki et al., 2009). On the other hand, roles for mTRAPP II and Rab11 (a Ypt31 homolog), together with the Golgi-to-PM regulators Rab8 and Rabin8 (a Sec2 homolog), were shown in human cells during primary cilia assembly (Westlake et al., 2011). These conflicting findings highlight the uncertainty regarding the roles of mammalian Golgi TRAPP complexes.

### Mammalian TrappC2/Sedlin

Mutations in *SEDL*, the gene that encodes Sedlin, were implicated in the cartilage disease SEDT (Table 1). Knockout of Sedlin/TRAPPC2 showed that the gene is not essential for cell viability (Table 2). Sedlin knockdown specifically affects export of procollagen, a large cargo that cannot fit into COPII vesicles, from the ER into megacarriers. While Sedlin probably does it in the context of TRAPP because mBet3 is also required for this process (Venditti et al., 2012), it is not clear which TRAPP complex is involved in this process.

### Mammalian TRAPP III

While there is currently no direct evidence for the existence of TRAPP III or for a role of TrappC8 in autophagy, TrappC8 was identified in a proteomic study as a component of the human autophagy interaction network (Behrends et al., 2010). Unlike yeast, based on genetic interaction maps and co-IP, a role for the mammalian TRAPP III was proposed in ER-to-Golgi transport together with COPII vesicles (Bassik et al., 2013). In addition, TrappC8 was shown to be required for cell entry of the human papilloma virus, implying its role in endocytosis (Ishii et al., 2013) and for ciliogenesis (Schou et al., 2014). Thus, the conservation of a role for TRAPP complexes in autophagy is still unclear.

### Plant TRAPPs

Plants have orthologs for all the TRAPP I and TRAPP II subunits essential in yeast and the TRAPP III-specific subunit Trs85 (Thellmann et al., 2010). As in yeast, TRAPP II was implicated in a late Golgi transport step together with the exocyst (Rybak et al., 2014). The functions of TRAPP I and TRAPP III in plants are currently unknown.

## TRAPP complexes as Ypt/Rab GEFs

### Golgi TRAPPs

Identification of GEFs for individual Ypt/Rabs is not straightforward because, although individual GEFs are conserved through evolution, they do not share obvious similarities with GEFs for other Ypt/Rabs. The implication of TRAPP as a GEF for Ypt1 was inspired by biochemical similarities observed independently in different labs between a purified Ypt1 GEF and the TRAPP complex (Jones et al., 1998; Sacher et al., 1998). Soon after, TRAPP was identified as a Ypt1 GEF (Jones et al., 2000; Wang et al., 2000). While the idea that TRAPP acts as a Ypt/Rab GEF is well-established, the Ypt-GEF specificity of individual TRAPP complexes has been under debate.

Using biochemical and genetic analyses, including GEF activity, co-precipitation assays, the effect of TRAPP mutations on Ypt cellular localization and high-copy suppression of mutations, we have shown that TRAPP I acts as a GEF on Ypt1 whereas TRAPP II is a Ypt31/32 GEF (Morozova et al., 2006). In contrast, based on lack of GEF activity of purified TRAPP II on Ypt31/32 (Wang and Ferro-Novick, 2002), inability of GST-tagged Ypt31/32 to pull-down a TRAPP II-specific subunit (Yip et al., 2010), and a proposed localization and function of Ypt1 at the late Golgi (Sclafani et al., 2010), the view that all TRAPP complexes act on Ypt1 throughout the Golgi was promoted (Barrowman et al., 2010). However, recently this view was disputed at multiple levels. First, the observed phenotypes of *YPT1* mutations assigning it a role in the late Golgi were shown to result from a defect in ER-phagy and not in late Golgi transport (Lipatova et al., 2013). Second, localization of Ypt1 and Ypt31 to opposite sides of the Golgi, early and late, respectively, was recently established (Kim et al., 2016). Third, the activity of TRAPP II as a GEF for the Ypt31 ortholog RabE^RAB11^ at the trans Golgi was shown in *Aspargilus nidulans*, a filamentous fungus, both *in vivo* and *in vitro* (Pinar et al., 2015). Therefore, the current model for TRAPP complexes and their Ypt substrates in yeast depicts that TRAPP I and TRAPP II activate Ypt1 and Ypt31/32, respectively, on the two ends of the Golgi (Figure 2).

Support for the current model comes from two metazoan model systems. First, in *Drosophila*, the Trs120 homolog *bru* was shown to function in the late Golgi, to interact with *Rab11*, a Ypt31/32 homolog, and to affect the latter's cellular localization (Robinett et al., 2009). Second, in *Arabidopsis*, TRAPP II was functionally linked to the Ypt31 plant ortholog Rab-A, but not to the Ypt1 ortholog Rab-D (Qi et al., 2011). These results support the idea that TRAPP II acts as a Ypt31/32 GEF not only in yeast. The mammalian Golgi TRAPP II (see above) was shown to act *in vitro* as a Rab1, but not Rab11, GEF (Yamasaki et al., 2009). While the GEF specificity of yeast and mammalian TRAPPs might differ, we suggest that more research is needed to resolve this issue.

### TRAPP III

In yeast, Ypt1 is required for stress-induced and the selective autophagy pathway CVT (cytoplasm-to-vacuole) (Segev and Botstein, 1987; Lynch-Day et al., 2010; Lipatova and Segev, 2012; Lipatova et al., 2013). The idea that the Trs85-containing TRAPP III complex activates Ypt1 in autophagy is based on the following evidence: First, Trs85 is required for bringing Ypt1 to PAS (Lynch-Day et al., 2010). Second, using bimolecular fluorescence complementation (BiFC), Ypt1 and Trs85 were shown to interact on PAS (Lipatova et al., 2012, 2015b). Third, overexpression of Ypt1 can suppress the autophagy defects of *bet3* and *trs85*Δ mutant cells (Lipatova et al., 2012; Zou et al., 2015). In addition to the BiFC interaction of Ypt1 and Trs85 on PAS, they also interact on Atg9 membranes. Once Ypt1 is activated on Atg9 membranes, it recruits Atg proteins to form PAS (Lipatova et al., 2012). Recruitment of two Atgs requires Ypt1, Atg1 and Atg11; only the last was shown to be a bona fide Ypt1 effector (Lipatova et al., 2012; Wang et al., 2013).

In mammalian cells, the orthologs of Ypt1 and Trs85, Rab1 and TrappC8, respectively, were implicated in autophagy (Behrends et al., 2010; Zoppino et al., 2010). However, because the similarity between Trs85 and TrappC8 is very low, and because TrappC8 was also suggested to play a role in ER-to-Golgi transport (Bassik et al., 2013), the connection of the mammalian TRAPP III complex to autophagy is currently not clear.

## TRAPP complexes as tethers

Tethers are proteins that can bridge two membranes and thereby capture vesicles (~500–1000 Å diameter) by an appropriate acceptor compartment. They are thought to bring two membranes into ~300 to >2000 Å proximity (Bröcker et al., 2010) and to facilitate vesicle uncoating and SNARE complex formation. SNAREs bring membranes to ~140 Å proximity and mediate membrane fusion (Hanson et al., 1997). Tethers can be long coiled-coil proteins or multisubunit-tethering complexes (MTCs), which typically interact with GTPases in their GTP-bound form, namely, they are downstream effectors of GTPases (Yu and Hughson, 2010).

While accumulation of vesicles when a putative tether is depleted agrees with the idea that this protein could be a tether, it does not prove this idea because the effect can be indirect or the protein might act in any sub step between vesicle formation and tethering, e.g., vesicle motility. Two types of assays have been used to establish tethering activity. First, an *in vitro* reconstitution assay was used to show that the HOPS complex is a tether (Hickey and Wickner, 2010). Second, an *in vivo* assay was used to show that directing the exocyst subunit Sec3 to the mitochondria, results in tethering of secretory vesicles to this compartment (Luo et al., 2014). Such assays have not been used for TRAPP yet.

The notion that TRAPP belongs to the MTC tether group was based on accumulation of ER vesicles in a cell-free system when TRAPP was depleted (Sacher et al., 2001). However, as mentioned above, this can result from an indirect effect, e.g., due to its GEF activity for Ypt1, which is required for recruitment of the Golgi tethers. If TRAPP is a tether, it is expected to bridge a distance >140 Å between two membranes. The length of TRAPP complexes ranges between ~180 Å for TRAPP I to ~250 Å for TRAPP II (Figure 1). Therefore, in principle, TRAPP complexes could act as tethers, especially in potential cooperation with other proteins. Like other known membrane tethers, TRAPP complexes interact with GTPases. However, unlike all other tethers, TRAPP complexes act as GEFs and not as downstream effectors of GTPases. The interaction of GEFs with their GTPase substrates is not stable and is interrupted once the GTPase binds GTP. Thus, it is unlikely that TRAPP GEFs and Ypts together create a bridge between two membranes. It is also not very likely that TRAPP GEFs cooperate with Ypt/Rab effectors to create membrane bridges, because the two bind to the two alternate forms of the GTPases, GDP, and GTP, respectively.

In yeast, the interaction of Bet3, a core TRAPP subunit, with Sec23, a component of the inner layer of COPII coat, was shown to be required for TRAPP binding to ER-derived vesicles (Cai et al., 2007). Interactions of TRAPP II and the non-essential TRAPP II subunit Trs65 with components of the COPI coat, Ret1/Cop1 and Gea2 (and Sec21), respectively, was used to implicate TRAPP II in tethering of COPI vesicles to the Golgi (Cai et al., 2005; Chen et al., 2011). In mammalian cells, it was suggested that TRAPP II interacts with COPI vesicles, the TRAPP II subunit TrappC9 interacts with a motor that moves COP II vesicle on microtubules, and COPII vesicles were suggested to interact with TRAPP III (Zong et al., 2012; Bassik et al., 2013). In addition to the inconsistency between yeast and mammalian cells, these findings provide information of TRAPP interactions with one membrane, while as a bridge it is expected to interact with two membranes. Interaction of the two Bet3 copies of a single TRAPP I complex with membranes of two separate vesicles was suggested to tether ER-derived vesicles during their homotypic fusion (Cai et al., 2008). However, the distance that one TRAPP I could bridge two membranes in this model is ~40 Å, which is shorter than a SNARE complex. Moreover, homotypic fusion of ER vesicles has not been shown in yeast, and the existence of TRAPP I in mammalian cells is questionable. Therefore, evidence that any TRAPP complex acts as a membrane tether is currently inconclusive.

To understand a possible mechanism by which TRAPP could act as a membrane tether, it is crucial to know how TRAPP complexes are positioned on membranes. Currently, the topology of TRAPP complexes on membranes is not known. TRAPP I was proposed to lie flat on one membrane through its two Bet3 copies, which would yield a distance shorter than expected for a tether. The idea that TRAPP I attaches to membranes through Bet3 was based on its hydrophobic channel (Kim et al., 2006), and/or palmitoylation. However, mutational analysis showed that neither is needed for TRAPP I function (Cai et al., 2008). The ways in which TRAPP II and TRAPP III are positioned on membranes are also unknown. Thus, additional evidence is needed to support the idea that TRAPP complexes act as membrane tethers and to elucidate mechanisms by which they might do that.

## TRAPP in disease

Because the secretory pathway is implicated in a broad range of human diseases (Aridor and Hannan, 2000), it was expected that it would be similar for TRAPP, a complex key for transport through the Golgi. Indeed, mutations in TRAPP subunits were linked to a wide range of human diseases (Brunet and Sacher, 2014) and their number keeps growing. Here, we highlight new disease connections (Table 1) and comment on the surprising fact that different TRAPP subunits seem to be linked to different diseases.

Examples of newly added disease connections for TRAPP subunits include miscarriage for TrappC2 (Wen et al., 2015), cancer for TrappC4 (Weng et al., 2014), and TrappC9 (Wetterskog et al., 2014; Zhang et al., 2015), intellectual disability and schizophrenia for TrappC9 (Khattak and Mir, 2014; McCarthy et al., 2014), and Alzheimer's disease for TrappC6 (Hamilton et al., 2011; Chang et al., 2015).

If there is really only one mammalian TRAPP complex (Barrowman et al., 2010), it is expected that mutations in individual subunits would have overlapping effects. Therefore, the most bewildering question about the involvement of TRAPP in human disease is why mutations in individual subunits result in such divergent diseases. One possible explanation is that there is more than one human TRAPP complex with different tissue specificities or cellular functions, and the different complexes are involved in different diseases. Another possible explanation is that “the screen is not saturated”; namely, the full range of representative diseases for individual TRAPP subunits has not been uncovered yet.

Recent findings connecting certain individual TRAPP subunits to multiple diseases support this idea. For example, only recently TrappC9 (Trs120) was connected to cancer and schizophrenia in humans (Khattak and Mir, 2014; McCarthy et al., 2014) and inflammatory disease in cows (Wang X. et al., 2015), thus broadening its disease connection from the previously known association with intellectual disability (Brunet and Sacher, 2014). Likewise, Alzheimer's disease was recently added (Hamilton et al., 2011; Chang et al., 2015) to the previously known association of TrappC6 (Trs33) with hypopigmentation (Brunet and Sacher, 2014). Another example is the addition of miscarriage (Wen et al., 2015) to the tissue specific disease X-linked SEDL associated with TrappC2 (Trs20) (Brunet and Sacher, 2014).

Recent findings also connect multiple TRAPP subunits to a single disease. For example, to date four TRAPP subunits are implicated in cancer: TrappC1 (Bet5), TrappC4 (Trs23) (Weng et al., 2014), TrappC9 (Zhang et al., 2015), and TrappC10 (Pongor et al., 2015). Likewise, two TRAPP subunits were recently implicated in brain-related diseases: TrappC6a (Trs33) (Hamilton et al., 2011; Chang et al., 2015), TrappC9 (Khattak and Mir, 2014; McCarthy et al., 2014).

Therefore, unless the disease divergence is due to moonlighting activities of individual TRAPP subunits unrelated to TRAPP, it is expected that association of individual subunits with more diseases will be uncovered and that some overlap between the different subunits, if not complete, will emerge.

## Summary and future questions

While plenty of information has been accumulated about TRAPP since its identification in yeast and mammalian cells and its coming to the limelight as a Ypt/Rab GEF, there are still multiple uncertainties and unanswered questions. Structure is known only for the small TRAPP subunits, but not for the large ones. Complex architecture is known for TRAPP I and to some extent for TRAPP III, but not for TRAPP II. It is also not clear whether dimerization of TRAPP II occurs *in vivo* and has a function. It is not known whether the cellular functions of the three known TRAPP complexes are conserved from yeast to human cells. The Ypt/Rab GEF specificity of TRAPP complexes is still under debate even in yeast. The topology of TRAPP complexes on membranes is not known and evidence for a direct role for TRAPP complexes in membrane tethering is lacking. Finally, the apparent divergence of human disease associated to different TRAPP subunits is unclear. Future experiments will hopefully help fill in these multiple holes in the current picture.

## Author contributions

All authors listed have made substantial, direct, and intellectual contribution to the work, and approved it for publication.

### Conflict of interest statement

The authors declare that the research was conducted in the absence of any commercial or financial relationships that could be construed as a potential conflict of interest.
